# A Review on Novel Applications of Nanoparticles in Pediatric Oncology

**DOI:** 10.32604/or.2025.069101

**Published:** 2025-11-27

**Authors:** Theano Makridou, Elena Vlastou, Vasilios Kouloulias, Efstathios P. Efstathopoulos, Kalliopi Platoni

**Affiliations:** 1 Pediatric Department, General Hospital of Veria, Veria, 59131, Greece; 2 Radiotherapy Department, General Children’s Hospital “Pan. & Aglaia Kyriakou”, Athens, 11527, Greece; 3 Department of Radiation Oncology, Medical School, National and Kapodistrian University of Athens, Athens, 11527, Greece; 4 Department of Applied Medical Physics, Medical School, National and Kapodistrian University of Athens, Athens, 11527, Greece

**Keywords:** Nanoparticles (NPs), nanomedicine, pediatric oncology, pediatric cancer diagnosis, pediatric cancer therapy

## Abstract

Nanomedicine has evolved significantly over the last decades and expanded its applications in pediatric oncology, which represents a special domain with unique patients and distinct requirements. Τhe need for early cancer diagnosis and more effective and targeted therapies aiming to increase the pediatric patients’ survival rates and minimize the treatment-related side effects to survivors is profound. Nanoparticles (NPs) come as a beacon of hope to provide sensitive cancer diagnostic tools and assist contrast agents’ transport to the malignant tumors. Besides, NPs could be designed to deliver targeted drugs and genes to tumors, minimizing the medicine-related toxicities. Metal and metal oxide NPs could be exploited as sensitizers to enhance chemotherapy and radiotherapy effects. Future research should emphasize pediatric models to gain secure results about NPs’ safety and efficiency for pediatric cancer patients. This review presents the recent studies on the use of NPs in pediatric cancer management and highlights their impact on diagnosis, treatment outcomes, and the quality of life of the survivors. The purpose of this study is to investigate the benefits that may arise from the use of NPs in pediatric oncology, address the potential limitations and challenges, and discuss the needs for future research efforts.

## 1 Introduction

Cancer is a leading cause of death for children and adolescents worldwide. According to the American Cancer Society, cancer is the second most common cause of death for children aged 1 to 14 years old and the fourth for children between 15 to 19 years of age in the United States [1]. It is estimated that each year, 400,000 children will be diagnosed with malignant tumors, while the technological advances in diagnostic applications and the novel therapeutic approaches have raised the 5-year survival to 80% and 30% in high-income and low-income countries, respectively [2,3]. Leukemia, brain and central nervous system (CNS) tumors, sarcomas, neuroblastomas, and lymphomas are among the most common cancer types for children up to 19 years old [4]. Pediatric cancer types differ significantly from those in adults as they are not associated with lifestyle or ageing, but rather arise from genetic mutations, developmental processes, or abnormalities in growing tissues. Hence, the divergence in tumor biology necessitates tailored approaches for pediatric cancer management.

Since pediatric cancer advances quickly and is more aggressive compared to adult, the early and accurate diagnosis is one of the most crucial aspects affecting treatment outcomes [5]. However, diagnostic radiology raises concerns in pediatrics. Firstly, in terms of diagnostic imaging, the lack of cooperation of the young patients leads to the use of sedation in order to acquire scans, ensuring both patient safety and high image quality. Not only the medications used for the sedation, but also the contrast agents, could cause either immediate side effects, such as allergy reactions, or long-term ones. The effect of these agents and drugs could increase significantly for patients who require multiple scans [6,7]. For instance, gadolinium (Gd)-based contrast agents have been found to form depositions in the brain, and they could cause significant damage to the kidneys of patients with chronic kidney disease [8]. Moreover, since the developing brain of the young patients is more vulnerable to damage compared to adults, research should focus on the long-term effects of the children’s brain depositions caused by the Gd-based contrast agents [9].

Regarding pediatric cancer therapy, the basic treatment options include or combine stem cell transplant, surgical resection of the tumor, chemotherapy, and radiotherapy (RT). However, chemo-and radio-resistance exhibited by cancer cells may undermine the effectiveness of the implemented therapeutic strategies and lead to subsequent tumor relapses. Apart from the conventional approaches, novel types of therapies such as gene therapies and immunotherapies are being developed constantly [10,11]. Yet, pediatric cancer therapy encounters certain difficulties. Organs and tissues in children develop rapidly, thus patients may exhibit developing-specific responses to cancer treatment. Besides, considering that mutational rates, pharmacokinetics, pharmacodynamics, and metabolic rates in the pediatric population differ greatly compared to adults, every product dosage must be adjusted to fit in pediatric cancer patients’ needs based on a broad range of parameters beyond just weight and age [12]. Even the form of the drugs has to be formulated to be appropriate for children and keep a taste that will be nice and attractive to them. In the case of brain tumors, the impermeability of the blood-brain barrier (BBB) is a significant obstacle [13]. Since BBB protects the nervous system microenvironment from pathogens or toxins, it limits the access of drugs to the brain as well [13]. Furthermore, the lack of adequate information regarding the pathophysiology behind common pediatric cancer diseases hinders the creation of effective, new drugs [10].

Chemotherapy and RT are associated with immediate adverse side effects such as hair loss, fatigue, nausea, diarrhea, anemia, etc. Besides, the daily administration of chemotherapy and the typical fractionation schemes in RT (5 fractions per week over several weeks) dictate the need for frequent clinic visits, which further impairs pediatric patients’ daily well-being. Despite the novel approaches in childhood cancer treatment, survivors may still suffer from treatment-related long-term side effects such as neuroendocrine problems, cognitive dysfunctions, hearing and vision problems, cardiotoxicities, nephrotoxicities, and fertility abnormalities. Moreover, compared to the general population, children who have been exposed to radiation from multiple CT scans or received RT for their primary cancer exhibit a higher risk of developing a secondary malignancy [14].

The aforementioned challenges in pediatric cancer treatment management highlight the need to optimize the diagnostic and therapeutic procedures according to the specific pediatric patients. Over the last years, nanoparticles (NPs) have conquered the research in a lot of domains of conventional medicine, including pediatric oncology [15,16]. Nanostructures emerge as a beacon of hope for the development of new targeted diagnostic and therapeutic methods and the improvement of the already existing ones.

NPs are tiny particles, the size of which ranges from 1 to 100 nm. They exhibit high surface-to-volume ratios [17], increased cellular uptake [18], and functionalization properties [19]. These properties render NPs ideal candidates for tumor targeting, drug transport, and sustained or controlled drug release, while the high atomic number (Z) of metal NPs could be exploited in order to enhance tumor radiosensitization in diagnostic imaging and cancer therapy [20,21]. As well, a great number of biosensors are currently under investigation, the technology of which is based on NPs [22].

The small size of NPs, combined with the enhanced permeability and retention (EPR) phenomenon, which occurs in solid tumors, suggests the passive tumor targeting where NPs could be allowed to pass through tumors and achieve adequate accumulation in their microenvironment. Due to their large surface area, NPs could also be functionalized with molecules, peptides, and antibodies in order to target the unique markers expressed in cancer cells and support the development of actively targeted drug delivery systems [11].

Depending on their composition, NPs are divided into three categories: organic, inorganic, and carbon-based [23]. Organic NPs, such as liposomes and dendrimers, which are biocompatible and biodegradable, could serve as drug carriers and vaccine components [23]. Moreover, they are characterized by an important sensitivity to heat and radiation, which could enable the controlled release of any drug under specific conditions [23]. Carbon-based NPs like carbon quantum dots (QDs) hold electrical conductivity and special properties regarding light and heat absorption [23]. Thus, combined with their low toxicity, they could be mainly used as imaging contrast agents and drug carriers [23]. Inorganic NPs include the rest of the NPs, such as metal-based and metal oxide NPs. Given their low toxicity, hydrophilicity, biocompatibility, easy surface functionalization, and stability, along with their tremendous electrical and magnetic properties, they could be used for biosensor engineering [22] and serve as radiosensitizers in imaging and therapeutic applications [24–26].

Even though thorough research exists in the field of NPs-aided cancer diagnosis and therapy for adults, studies in pediatric cancer are limited. This review provides an overview of the current and most recent research around the applications of NPs in pediatric oncology services.

## 2 NPs in Pediatric Oncology

### 2.1 Pediatric Cancer Diagnostic Imaging

Ferumoxytol, an iron oxide (FeO) NP with a hydrodynamic diameter of 17–31 nm, is already approved as an iron supplement by the Food and Drug Administration (FDA) [6,27]. Its core consists of FeO, and its coating is made of polyglucose sorbitol carboxymethyl ether [27]. It is biodegradable, biocompatible, and has a long intravascular half-life time of approximately 14 h [6]. Additionally, ferumoxytol exhibits the potential to accumulate in the reticuloendothelial system (RES) [6,27,28]. It is administered by intravenous (IV) infusion [29]. Its use as a contrast agent has application in approximately every system, from metastases detection to pathologies of the cardiovascular system. Until now, it has been used off-label in children as a contrast agent in Magnetic Resonance Imaging (MRI) [6,30].

The long intravascular circulation time of ferumoxytol, especially in the pediatric population, gives the opportunity for breaks and extra time to calm the patients down and help them cooperate during a diagnostic imaging procedure [6]. In this way, the need for anesthesia may be reduced, leading to a lower cost of the examination and fewer side effects from the sedative medication. The feed and sleep method can also be applied. In this method, the contrast agent is injected into patients before imaging, and then they go to sleep [6]. The examination is initiated only when the patient is asleep [6]. Even if the procedure is disrupted, there is enough time to continue without the need for a new injection [6].

Regarding its safety, it requires an intact iron metabolism to be administered. Although small doses of 5 mg/kg do not lead to iron accumulation, its main contraindication includes patients with hemosiderosis and hemochromatosis, where iron overload pre-exists [9]. At the same time, it is safe for patients with chronic kidney disease, and it does not cross the BBB [9].

#### 2.1.1 Imaging in Brain Tumors

CNS malignancies hold first place regarding the most common solid tumors in children [31]. Diagnostic imaging results and stage classification are of critical importance, since they determine the therapeutic scheme that should be followed for the patient’s treatment (e.g., surgical resection, chemotherapy, and/or RT, etc.). The diagnostic imaging of brain tumors is degraded by the presence of the BBB. The BBB forms a side-dependent barrier, mimicking a protective wall for the brain by prohibiting the entrance of non-essential molecules. It consists of tight junctions between the endothelial cells, the basement membrane, astrocytes, mural and immune cells [13]. However, positively charged lipophilic molecules can overcome the BBB, whereas the transport of other essential molecules is facilitated by specific membrane carriers [15]. Efflux transporters are part of the BBB and hinder the entrance of small lipophilic molecules into the brain [32]. NPs can overcome the obstacle that efflux transporters create by facilitating a receptor-mediated transport through specific pumps [32]. Those pumps include insulin, transferrin, and low-density lipoprotein receptors. NPs’ membrane can be formulated with the appropriate molecules, in order to achieve the crossing of the BBB [32].

The Gd-enhanced MRI constitutes the gold standard for the diagnosis and staging of brain tumors [33]. Nevertheless, the low permeability of the BBB decreases the sensitivity of the MRI and, in some cases, MR images fail to detect cancer in its early stages or to fully depict the tumor and its margins. Additionally, Gd depositions have been found inside the brain parenchyma of patients who underwent multiple scans [8]. Taking into consideration that children are still developing organisms, a lot of concerns arise regarding the Gd effect on their neurocognitive function. In addition to that, its inability to successfully characterize the margins of the tumor creates an important obstacle in some types of fast-growing malignancies, where the exact limits have to be determined for the optimum surgical resection [15].

Despite the progress in glioblastoma (GBM) diagnosis and treatment, it is still associated with low survival rates [31]. Investigating GBM imaging, Covarrubias et al. proposed the use of NPs to target the Receptor protein tyrosine phosphatase mu (PTPmu), which can be found both at the tumor itself and at a distal location of it [31]. FeO formed into NP chains was conjugated with the SBK2 peptide, which is the one that binds to the PTPmu. The SBK2-FeO NP was used as a contrast agent for MRI imaging. For the purpose of the experiment, the team developed a pediatric GBM model using athymic mice to place SJ-GBM2 cells from a 5-year-old patient orthotopically. Liposomes conjugated with the SBK2 peptide and fluorescence were used for optical imaging and histology examination. Their experiment included both *ex vivo* and *in vivo* sections. This model successfully exhibited the special characteristics of the pediatric GBM. The T2-weighted MR images, which were received from the athymic mice with the SJ-GBM2, demonstrated that PTPmu conjugated NPs accumulated both inside the tumor with its margins and also inside the individual GBM cells that had migrated millimeters away from it. The successful targeting of both proximal and distal GBM cells using PTP mu-NPs opens the road to theranostic applications for GBM in pediatric patients.

#### 2.1.2 Imaging in Metastases and Malignant Lymph Node (LN) Detection

For cancer staging and treatment scheme planning, the metastases and LN detection are a critical step. In general, LNs larger than 1 cm are characterized as malignant. This assertion, when applied to the pediatric population, shows low sensitivity. LNs reacting to an infection can also be larger than 1 cm, and LNs smaller than 1 cm can potentially be malignant [34]. Hence, LN size on its own cannot differentiate the benign and malignant ones. To date, the test of choice for the detection of malignant LNs and metastases is an ^18^F-fluorodeoxyglucose (^18^F-FDG) enhanced positron emission tomography (PET) scan. Even the results of PET may be affected by the reaction of the LNs to an infection [28].

The research team of Anne Monika Muehe and Florian Siedek conducted a trial (NCT01542879) to evaluate the use of ferumoxytol as a contrast agent in PET/MRI to distinguish malignant and benign LNs in children diagnosed with cancer [28]. During the trial, all the participants underwent integrated ^18^F-FDG PET/MRI scans. The patients received two scans, six months apart. The LNs that remained the same in both scans were characterized as benign, and the ones that changed were characterized as malignant. A histology evaluation took place only on samples received from two patients, where the solid tumor and the neighboring LNs were surgically resected. The enhanced imaging of the hilum was the criterion for the characterization of a LN as benign or malignant. More specifically, the absence of ferumoxytol uptake was indicative of malignancy. The team came to the conclusion that ferumoxytol-enhanced PET/MRI has high sensitivity regarding the characterization of malignancy in LNs between 5 and 10 mm. Besides, ferumoxytol was well tolerated by the patients without significant side effects.

#### 2.1.3 Imaging in Liver, Bones, and Soft Tissue Tumors

The tiny size of NPs provides the EPR effect in cancerous tissues, providing useful information for the differential diagnosis of benign and malignant tumors [35]. Additionally, NPs accumulation at the RES allows an accurate demonstration of tumors at the liver, spleen, and bone marrow, by producing a low signal at T2-weighted images (T2WI) [6]. Regarding bone and soft tissue tumor imaging, ferumoxytol creates a more intense contrast between the healthy and cancerous tissue, thus it provides enhanced image quality compared to Gd [6]. Additionally, neoplastic bone marrow cannot take up ferumoxytol, which could assist with the differentiation between healthy bone marrow and metastatic lesions.

The adult bone marrow presents with a fatty conversion, leading to easier detection of the metastases [27]. On the contrary, children’s bone marrow still possesses a great number of hematopoietic cells, which are hardly distinguished from the metastases on MRI scans [27]. Nevertheless, bone marrow is an organ of the reticuloendothelial tissue, which means that the leakage and the subsequent phagocytosis of ferumoxytol from the macrophages also takes place there, producing a negative signal in T2-weighted MRI [27]. The focal metastatic sites of the bone marrow do not take up ferumoxytol and subsequently do not produce the negative signal in T2-weighted MRI, providing a great tool for the detection of metastases [27].

On this ground, Rashidi et al. compared the sensitivity of ferumoxytol-enhanced MRI to the detection of bone marrow metastases in children and young adults [27]. All patients were between 2 and 25 years and had been diagnosed with both extracranial solid tumors combined with one or multiple bone marrow metastases. The MRI scans were repeated after chemotherapy was applied. The results revealed the increased sensitivity of the enhanced MRI (99% of the metastases detected) against the unenhanced (94% of the metastases detected). An interesting finding was the higher sensitivity of the post-chemotherapy enhanced MRI, attributed to the increased hematopoietic activity of the bone marrow. The higher sensitivity led to a better depiction of the tumor size and its metastases, enabling the construction of a more efficient treatment approach.

Liver tumors, and especially hepatoblastoma, which is the most common, are well depicted by ferumoxytol-enhanced MRI, providing a detailed description of the tumor, its margins, and its vascularization [6]. Focal nodular hyperplasia (FNH) represents a benign liver tumor, where ferumoxytol can assist with the diagnosis. In this case, Kupffer cells inside the FNH are responsible for the ferumoxytol uptake, leading to signal loss in T2 and T2* weighted images. This signal loss provides an important diagnostic tool to assist in distinguishing FNH from the malignant lesions [6].

#### 2.1.4 Imaging for Sarcoma Staging

Tumor margins, the neurovascular involvement, and tumor thrombi are among the most crucial parameters upon which the sarcoma treatment scheme is based [9]. Siedek et al. compared the sensitivity of Gd and ferumoxytol-enhanced MRI regarding the depiction of bone and soft tissue sarcomas in the pediatric population [9]. The results showed that both contrast agents could assess the margins and the size of the tumor without a significant difference. Nevertheless, ferumoxytol provided more accurate information for the neurovascular involvement and tumor thrombi, suggesting that ferumoxytol could be used as a Gd substitute adequately.

#### 2.1.5 Imaging for the Detection of Joint and Pleural Infiltration

Theruvath et al. conducted a pilot study regarding the depiction of joint and pleural infiltration of bone sarcomas in young patients [35]. Their study was based on the fact that malignant tissues produce exudate due to the increased permeability of the microvascular system of the synovium. As a result, not only small particles, but also larger ones (like ferumoxytol) can accumulate in the fluid. On the contrary, the reactive fluid caused by malignant tissues, which are found neighboring the healthy joints, does not contain NPs. The microvascular structure of the joints, which the tumor does not infiltrate, is maintained and does not allow the larger NPs to cross it. The MRI scans, which were carried out 1 h after ferumoxytol infusion, showed no difference in signal between the healthy and the cancerous tissue, while the ones taken 24 h later demonstrated a significant enhancement of both the joints and pleural effusions in T1 MRI images of patients with tumor infiltration. At the same time, no or minimal difference in enhancement was noted in patients without spreading disease and in those with benign tumors. This phenomenon was explained by the longer time that NPs required to accumulate at the site. The longer t1/2 time enables the scans to be taken 24 h post-infusion, providing the essential time space for ferumoxytol accumulation [36].

Evidence has shown that ferumoxytol-enhanced MRI can be utilized as a tool for a detailed characterization of the tumor margins around joints and the chest wall. Especially regarding joints, accurate imaging and precise diagnosis are of great importance, because of the different surgical resections that a spreading disease requires and its further impact on the daily life of a young patient [37].

### 2.2 NPs as Components of Biosensors

Sensors are devices that detect molecules in a variety of samples, such as blood and pharyngeal swabs, stool, or urine. The detection tests can be conducted close to the patient, and no special equipment is needed. In addition, test results can be assessed immediately. In the pediatric population, sample collection is usually a complicated process, and the sample amount is less than that of adults. NPs, due to their special properties, are suitable prospects for the design of sensors. Their surface can be formulated and linked with antibodies or other molecules in order to bind with the desired substance. AuNPs have already been used for such applications [38].

#### 2.2.1 Immunosensors for the Diagnosis of Adrenocortical Adenoma in Pediatrics

Pediatric adrenocortical adenoma is a rare and aggressive type of childhood cancer [39]. The excessive secretion of dehydroepiandrosterone sulfate (DHEAS) is its main feature. The lack of symptoms at the first stages of the disease complicates the diagnostic procedure, while the high aggressiveness of the later stages decreases the survival rate [40]. A key component of an early diagnosis would be the detection of DEHAS in plasma samples. Lima et al. engineered an immunosensor with AuNPs, which is able to detect DEHAS in blood samples from children with great sensitivity and specificity [39]. The AuNPs were functionalized with L-arginine (AuNPs-ARG) and connected with anti-DHEA IgM antibodies (AuNPs-ARG/IgM) to detect and bind with the DHEAS. The AuNPs-ARG/IgM were later attached to the oxidized glassy carbon electrode, creating the immunosensor ox-GCE/AuNPs-ARG/IgM. AuNPs-ARG amplified the signal received when DEHAS was connected with the IgM [39]. The clinical samples tested on the sensor were also tested with the electrochemical impedance spectroscopy, with similar results, leading to the conclusion that the immunosensor poses a great alternative to the detection of DEHAS with a limit of detection of 7.4 μg/dL.

#### 2.2.2 NPs as Components of Arrays for the Diagnosis of Acute Leukemia in Children

Leukemia is the most common malignancy in pediatric oncology [41]. The diagnostic strategy involves blood sample microscopy followed by bone marrow biopsy or aspiration [41]. A delayed diagnosis, especially in children, is attributed to its non-specific symptoms that can mimic common and usually non-life-threatening pathologies in young patients [41].

Volatile organic compounds (VOCs) represent carbon-based macromolecules produced by the cell metabolism and excreted to body fluids such as blood, breath, and feces [41]. The type of VOCs varies greatly and depends on various factors. Cancer cells’ metabolism is forced to adjust to the increased needs of the rapidly multiplying rate [42]. As a result, the type of VOCs found on cancer cells differs from the ones found on healthy cells, as shown in Table 1 [42]. Some VOCs are released exclusively by healthy cells or by cancerous tissues, while some of them are produced by both types at different concentrations.

**Table 1:** The different types of VOCs are categorized based on the type of cell population that produces them [42]

Healthy cells exclusively	Cancer cells exclusively	Both types of cells
Methyl sulfide	Hexanol	2,4dimethylheptane
4-methyldecane	Cyclohexanol	Benzaldehyd
		o-xylene
		Hexanal
		Methylbeneze
		Hexadecane
		3,7dimethyldodecane
		Chloroform
		Ethanol

Note: VOCs, Volatile organic compounds.

VOCs are mainly detected and analyzed by gas chromatography, which is an expensive and time-consuming method that requires special equipment [43]. A sensor called an E-nose was engineered to detect the VOCs easily and rapidly. Bordbar et al. utilized silver NPs (AgNPs) and AuNPs to create an array capable of recognizing VOCs from healthy individuals and patients with leukemia [41]. The sensor detected the blood vapor using 16 different NPs modified with eight different agents so as to be able to aggregate in the presence of a specific VOC and create a pattern for each sample.

The experiment was conducted using blood samples from patients between 2 and 18 years old, who were already diagnosed with leukemia, with either aspiration or biopsy of the bone marrow [41]. Healthy participants were also examined by a pediatric oncologist before sample collection. Cancer patients had received neither chemotherapy nor RT as part of their therapeutic scheme. The presence of specific VOCs in blood samples caused the aggregation of the NPs and resulted in the color change on the assay and the creation of a different pattern. The appropriate conditions regarding temperature and the time required for the color change were also investigated during the experiment (60°C for 4.5 h). After the formation of the patterns, the arrays were used for principal component analysis by computer-based algorithms. The patterns of the leukemia patients differed greatly from those of the healthy ones. The specificity, sensitivity, and accuracy that arose from the experiment were really impressive, showing that the sensor could detect VOCs in patients with leukemia, but not distinguish the different types of leukemia. While this study indicates the advantageous use of NPs to gain a fast, inexpensive, and accurate screening for leukemia, further investigation is required in order to examine the arrays that hold the potential to identify the different leukemia types and evaluate treatment response.

Table 2 summarizes the discussed studies in pediatric cancer diagnosis supported by NPs and their advantageous characteristics that support their implementation in diagnostic procedures.

**Table 2:** NPs in pediatric cancer diagnosis

Type of NPs	Application	Advantages
Ferumoxytol-FeO NPs	Contrast agent for MRI [6,9,35,44]	Biodegradability
		Biocompatibility
		Higher sensitivity compared to Gd
	Glioblastoma MRI depiction [24]	Theranostic Applications
AuNPs	Immunosensors for the diagnosis of adrenocortical adenoma [39]	Rapid results
AgNPs and AuNPs	Array for the detection of acute leukemia [41]	Rapid result
		Lower Cost

Note: NPs, Nanoparticles; FeO, Iron oxide; AuNPs, Gold Nanoparticles; AgNPs, Silver Nanoparticles; MRI, Magnetic Resonance Imaging; Gd, Gadolinium.

### 2.3 Pediatric Cancer Treatment

A great variety of anti-cancer treatment options, including chemotherapy, RT, and surgery, are currently implemented in pediatric oncology. Nevertheless, due to the unique characteristics of childhood tumors, the treatment approaches appear less effective against them in comparison with the same types of adult malignancies [16].

The impact of chemotherapy on healthy organs includes direct toxicity, liver metabolite-induced indirect toxicity, immune system suppression, reduced oxygen supply, inflammation, etc. [44]. In order to minimize such treatment-related effects, the dose of chemotherapy is often limited, reducing the potential efficacy of the treatment. Concerning RT, although higher dose prescriptions could improve tumor control, the tolerated dose of the healthy, developing, radiosensitive organs lying adjacent to the tumors restricts any further increase. Depending on the irradiated site, acute and late toxicities related to RT could include skin wounds, spine growth deficiency, hearing loss, eye damage, xerostomia, dysphagia, diarrhea, etc. Moreover, permanent damages from the tumor resection, such as cognitive problems, endocrine imbalance, developmental and neurological disorders, etc., could result in lifelong complications for the survivors [45,46]. Thus, the need to develop novel and targeted therapies for young cancer patients is apparent.

Nanomedicine provides some useful tools to increase treatments’ efficacy and decrease treatment-related side effects. Firstly, NPs could facilitate targeted treatments either by exploiting EPR or by adjusting them to target specific cells and tissues. Tumor targeting could introduce more efficient and less toxic therapeutic approaches. Besides, NPs have the desired size and potential hydrophobic structure to overcome the BBB, while NPs’ surface could be modified using various markers in order to bind with pumps and achieve drug delivery across the BBB [47]. Moreover, NPs have introduced the stimulus-responsive treatment, which is based on their ability to act under the surveillance of either innate stimulus (tumor microenvironment conditions like pH and temperature) or external stimulus (e.g., heat, radiation) [48]. Metal and metal oxide NPs embedded in tumor regions have been shown to enhance the radiation dose and increase tumor growth inhibition upon irradiation [49,50].

#### 2.3.1 Gene Therapies

Small interference RNA (siRNA)

During the creation and development of a malignant cell population, a great variety of signaling pathways are activated, and at the same time, the repairing and apoptotic mechanisms are being suppressed or deactivated [16]. The modification of these pathways and mechanisms at the gene level could significantly improve the therapeutic outcome. Regarding cancer treatment, siRNA is mainly used in order to silence a variety of genes, leading to the modification of signaling pathways [21]. A great obstacle to these types of therapy is the instability of the nucleic acids and the low permeability of the BBB when it comes to CNS tumors. The encapsulation of the nucleic acid in NPs and the functionalization of the NPs membrane with the proper molecules would probably lead to a more targeted treatment and also assist in BBB bypass [16].

Abballe et al. designed AuNPs, which would encapsulate siRNA against the Ape 1 gene to treat pediatric Ependymoma and MB cells [16,51]. Ape 1 is the gene responsible for the expression of an enzyme that repairs the damage caused by the RT [16,51]. The silencing of such a gene would increase the sensitivity of the tumor to RT [51]. The engineered AuNPs were coated with polyethylene glycol (PEG) and contained positively charged polyethyleneimine, which assisted with the binding of the negatively charged nucleic acid. At the same time, chitosan enabled the covalent binding of PEG and polyethyleneimine with the NPs. The results indicated the stable binding of the siRNA combined with a successful transfer and uptake by the cancer cells. The silencing of the gene led to lower expression of the Ape 1. Combined with photon irradiation, significant damage to the DNA of the cancer cells and activation of the cancer cells’ apoptotic mechanisms were achieved. Both studies hold great promise for this radio-resistant type of cancer.

Another promising formulation of Lipid NPs (LNPs)-siRNA was designed to treat pediatric Acute Myeloid Leukemia (AML) [52]. Recently, light was shed on the role of the non-coding genes and RNAs in the regulation of gene expression, especially those associated with cancer cell cycle, and their relevance to AML [52]. A non-coding gene, with a possible oncogenic role in AML, was recently found in the t (8;21) translocation [52,53].

Connerty et al. identified the exact non-coding sequence LINC01257 and investigated its correlation with AML, to demonstrate its oncogenic profile and its relation with worst prognosis and poorer therapeutic outcomes [53]. On this ground, the team designed a formulation, which consisted of an LNP as a vector and a siRNA to target the long non-coding RNA (lncRNA) region LINC01257. LNPs represent the most well-known and tested vehicles for nucleic acid transfer [53]. Cell cultures from children with AML were used for the study. The results demonstrated the specificity of the oncogenic lncRNA LINC01257 for AML, due to the fact that it was not expressed in healthy tissues. This study’s findings showed the positive effect of silencing, providing a possible therapeutic approach against pediatric AML. Additionally, the formulation achieved an impressive uptake rate, and no significant toxicity was observed in the healthy cells. Nevertheless, more research in animal models has to be conducted to evaluate the results thoroughly.

DNA therapies

Poly (beta-amino ester) NPs (PBAE) represent a conventional and promising nanocarrier for nucleic acids [54]. They are polymeric NPs with a high ability to encapsulate DNA and transfer it inside the cell, avoiding the endosome and leading to the expression of the carried genes [54]. Additionally, their rapid degradation decreases their potential toxic effects [54]. They exhibited great results when tested on adults for cancer treatments, causing minimal side effects [54]. At the same time, their good immunogenic and degradation profile makes them suitable candidates for the treatment of childhood cancers.

Choi et al. based on the positive results received from the encapsulation of the DNA responsible for the encoding of the herpes simplex virus type I thymidine kinase (HSVtK), at the adult glioma, constructed a PBAE NP to transfer the same DNA to treat atypical teratoid/rhabdoid tumors and MB in athymic mice with orthotopic tumor xenografts, consisting of pediatric brain cancer cells [54]. The plasmid DNA was encapsulated effectively inside the PBAE NPs due to the presence of hydrolytically cleavable ester bonds. Different types of polymers were used to determine which one of them has the best results in each type of tumor. Initially, NPs uptake was tested *in vitro* in cultures from pediatric brain cancer cells, and later ganciclovir was added, in order to activate the cell killing. When tested *in vivo*, NPs were administered by conventional enhanced delivery (CED) (direct delivery inside the brain parenchyma, through intracranial microcatheters), while ganciclovir was delivered intraperitoneally. The results revealed a significant increase in the survival of the treated population. A notable observation was that even minimum differences in the NPs structures can affect the uptake and transfection process, leading to the conclusion that each type of polymer had a different impact depending on the type of tumor. Although Poly(beta-amino ester)s (PBAEs) are very promising as DNA vectors for gene treatments in pediatric cancers with low toxicity and minimized side effects, more studies have to be conducted to determine the uptake mechanisms and pathways.

#### 2.3.2 Immunotherapy

Children’s immune system differs greatly from that of adults. From birth until adulthood, it is subject to changes, going through different stages, which vary greatly both in functionality and maturity. Hence, the immunotherapies already designed and approved for adult patients may lack efficiency in the pediatric population. Consequently, it is necessary to design novel ones, tailored for the developing immune system of the young patients [15].

Malignant cells are able to either deactivate the immune system or hide from it by expressing markers found in healthy cells [15]. Immunotherapy refers to the redirection of the immune system against the tumor and its microenvironment [15]. This can be achieved either by reactivating the immunity mechanisms or by targeting malignant cells, using antigens on the NPs’ surface [15]. NPs provide perfect vehicles to safely transfer molecules to specifically target cancer cells and activate an immunological response towards them.

Mendez-Gomez et al. designed an LNP able to carry different kinds of mRNAs to treat pediatric gliomas [55]. The LNP contained specific 5^′^ and 3^′^ untranslated regions that were able to incorporate into either personalized mRNA or the H3K27M mRNA, which has been associated with diffuse midline gliomas. The results showed a significant anti-tumor immunological response in murine brain tumors. In this study, NPs exhibited the ability to include other mRNAs for co-delivery. Toxicity studies proved its safety, leading to the obtainment of FDA-IND approval for human trials in the pediatric population with high-grade gliomas (NCT04573140) [15,55].

To target a specific cancer cell population and create an immune response against it, it is necessary to find markers expressed only in malignant cells; otherwise, the healthy cells will also be targeted, and the immune response will be massive, creating an inflammatory environment and causing damage to healthy tissues [15]. The stimulator of the interferon genes (STING) represents a pathway, greatly expressed in cancer tissues and especially in neuroblastoma cells [48]. This offers the opportunity to activate the intrinsic immune mechanisms against the tumors and their microenvironment, creating a T-cell-mediated response that could lead to cell death [55].

Neuroblastoma is characterized by a highly immunosuppressed population of cells, while the infiltration of T-cells is really low [56]. The team of Wang-Bishop exploited the STING pathway to treat neuroblastoma in mouse models [56]. To activate it, they utilized the 2^′^3^′^-cGAMP, which is a natural ligand and presents with high affinity for the pathway. As a vehicle, they used a PEG-coated polymersome to enter the 2^′^3^′^-cGAMP inside the cell. When the NPs entered the endosome, due to the low pH of the latter, the polymersome disassembled, while at the same time it mediated the transfer of the 2^′^3^′^-cGAMP to the cytosol. The NPs were directly administered inside the tumors. The activation of the STING pathway was tested in neuroblastoma cells both with and without N-MYC amplification, with similar results. N-MYC is an oncogene whose amplification is associated with neuroblastoma. The activation of type 1 interferon and other proinflammatory cytokines was massive, while the concentration of T-cells in the cancerous microenvironment was increased. In addition to that, cell death was activated, leading to the recession of the tumor growth. The created immunological memory could provide protection against a potential tumor relapse. This could open the road for immunotherapy in children suffering from neuroblastoma.

#### 2.3.3 Drugs

The most traditional loads of NPs are conventional drugs, which constitute the active ingredient of the final medicinal product. In most cases, the active ingredient shows high toxicity, causing systemic side effects when administered alone. Additionally, some anticancer drugs are not capable of crossing the BBB [57]. NPs utilize receptor-mediated endocytosis to get through the BBB. Insulin, transferrin, and endothelial growth factor are the most common receptors used as vectors to mediate the crossing of the BBB [15,16].

The drugs that exhibit great results regarding their efficiency against cancer cells may lack in pharmacokinetics, since they either degrade rapidly when entering the body or lack stability [57]. As a result, the amount of the medication that reaches the tumor is really low in comparison with the administered. Consequently, the anticancer effect is reduced, while the side effects remain the same. The encapsulation of drugs in NPs could increase the bioavailability of the medicine, while NPs labelled with targeting moieties could ensure adequate cellular uptake.

This could be described in the case of N(3)-propargyl, which is a derivative of temozolomide (TMZ) and a great candidate against diffuse intrinsic pontine glioma (DIPG) in the pediatric population [57]. N(3)-propargyl could bypass the mechanisms that are responsible for the resistance of the DIPG to TMZ and act against the cancer cells. Nevertheless, the pharmacokinetic properties of N(3)-propargyl hinder its use, due to its instability at normal pH and its inability to cross the BBB. To overcome this pharmacokinetic obstacle, two NPs were proposed. The first one is an apoferritin (AFt) nanocage, which targets the abundant transferrin receptor 1 found on the cancer cells, while its pH dependency allows the release of its content only inside the lower pH of the malignant microenvironment. The second NP included a sulfobutyl ether β-cyclodextrin complex transferred inside a liposome. Cyclodextrin was used in order to enhance the encapsulation of insoluble drugs into the aqueous core of the liposome and to prevent both leakage of the drug and its interaction with the membrane. Both complexes achieved good loading and were administered with CED directly to the rats’ brains, bypassing the BBB. Both of them provided good pharmacokinetics with sustained release and stability. Especially, the exploitation of transferrin receptor 1 as a target led to a great amount of drug entering the tumor cells. The combination of NPs and CED could provide new insight into the treatment of a variety of intracranial moieties, but the need for more preclinical trials to fully understand its mechanisms is imperative.

Infante et al. developed a micelle NP loaded with Glabrescione B to treat H-dependent MB [58]. Glabrescione B is a hedgehog inhibitor with poor water solubility, which negatively affects its pharmacokinetics [58]. The micelle is an amphiphilic self-assembled and improves the pharmacokinetics and solubility of the complex, which displayed minimum toxicity. For the purpose of the experiment, MB cultures were used for the cytotoxicity and cell proliferation studies. Mice with orthotopic allograft models of H-dependent MB were employed for the *in vivo* evaluation of the nanoassemblies. The administration of the medicine resulted in a significant decrease in the growth of the tumor.

Yang et al. used NPs to combine drugs to treat neuroblastoma in mouse models [59]. The vehicle was a liposome, loaded with picoplatin and cantharidin. Picoplatin is a very strong chemotherapeutic agent, but it is only active in its II form, which is also extremely toxic. Hence, it was encapsulated as picoplatin prodrug (IV form) to be converted to its active II form inside the cancer tissue. The results indicated a significant reduction in the tumor size, with really low levels of toxicity [59].

Liyanage et al. synthesized NPs containing Gemcitabine (GM) and iron transferrin protein (Tf) [60]. GM is an anti-cancer agent, leading to cell death by inhibiting the synthesis of DNA through DNA polymerase, whose activity is being blocked. Carbon Nitrate dots (CNDs) were used due to their water solubility, low toxicity, biocompatibility, high photoluminescence, and their adjustable structure. In order to overcome the BBB, the team utilized the Tf to take advantage of the receptor-mediated endocytosis. The abundance of Tf receptors in cancer cells enables a more targeted therapy. Firstly, the CNDs were synthesized and conjugated with GM. Later, transferrin was added by forming carbodiimide crosslinking. Then the NPs were characterized in order to define their physicochemical properties and ensure the attachment of both GM and Tf to CNDs. The efficacy and anticancer effect of the medicinal product were examined by testing GM alone and GM-CNDs on pediatric cancer cell cultures. Although the anticancer effect of the GM-CNDs was satisfying, the neighboring cells endured some damage. To overcome this, the targeting ability of Tf receptors was utilized. The combination of CNDs-GM and Tf resulted in the elimination of only the cancerous tissue, while the neighboring healthy tissue was not affected. Thus, the targeting capability and the anticancer effect of the system were established. The next step was to ascertain the ability of both the CNDs alone and the conjugated form to cross the BBB in zebrafish. CNDs achieved sufficient uptake/accumulation in the zebrafish brain. This finding was attributed to CND’s small size and specific structure. Another possible explanation is the exploitation of the transporter-mediated channel, which was achieved by mimicking the glutamine structure. The study indicated that the Tf not only enhanced a more targeted treatment but also improved the BBB crossing and ensured that the medicine reached the pathological cells.

In order to bypass the skepticism around the use of NPs as vectors for medicines in pediatric cancer therapy and overcome the loading and release problems, Rodríguez-Nogales et al. created a nanomedicine by combining two already known drugs with squalenic acid (SQ) [61]. SQ represents a biocompatible lipid molecule with the ability to self-assemble when connected with the drug in order to create stable nanostructures [61]. The active substances that were used were GM and edelfosine. The anticancer effect of the multidrug was tested on cell cultures derived from osteosarcoma metastasis of pediatric patients [62]. At the same time, its efficacy was compared with that of the squalenoyl-gemcitabine (SQ-GM) and SQ-GM-free edelfosine. The results demonstrated the superiority of the multidrug by presenting a better antiproliferative effect in comparison with either SQ-GM or SQ-GM with free edelfosine. At the same time, the anticancer effect was reinforced due to the synergistic effect of the two drugs. The formulation also displayed its anticancer effect at a lower concentration of its compounds, leading to lower toxicity. The pharmacokinetics were investigated *in vivo* in athymic mice. The IV administration was successfully achieved without the hemolytic effect of edelfosine, while at the same time, the formulation led to a sustained release of GM, significantly decreasing its toxicity. Overall, these multidrug nanoassemblies provided a potential alternative for the treatment of aggressive pediatric metastatic cancers.

In the field of pediatric hematology, drug-loaded NPs have been tested against AML [63]. Carvalho et al. designed polymeric NPS, which were formulated to target the CD33 transmembrane protein, a common marker on AML cells. ABT-737 and Purvalanol A were encapsulated inside the NPs. ABT-737 is an inhibitor of the antiapoptotic B-cell lymphoma (Bcl) proteins Bcl-2, Bcl-w, and Bcl-xL. Purvalanol A blocks the binding of cyclin-dependent kinase 1 and 2, disrupting the cell cycle. The effect of the NPs was tested on pediatric AML cells. The results demonstrated the synergistic effect of the two drugs and the induction of the AML cells’ apoptotic mechanisms.

#### 2.3.4 NPs as Sensitizers for RT and Photothermal Therapy

Numerous studies over the last decades have elaborated on the potential benefits of NPs-aided RT [64]. Metal and metal oxide NPs have been widely explored in the literature *in vitro*, *in vivo*, and *in silico* for their potential to serve as tumor radiosensitizers [65–67]. The difference in mass-energy absorption coefficients between metal and soft tissue indicates that the presence of high-Z NPs in an irradiated tumor volume could enhance the intratumoral dose deposition and consequently amplify reactive oxygen species (ROS) production, DNA damage, and cancer cells apoptosis. Since the dose enhancement seems to be restricted in short radial distances from the NPs aggregation area, healthy organs adjacent to tumor volumes would not be furtherly affected. The majority of studies investigate the radio-sensitization potential of AuNPs, AgNPs, and Gd-based NPs in external beam RT and brachytherapy. Gd-based NPs, along with the NBTXR3, which is a hafnium Oxide (HfO) NP, are employed in most of the conducted clinical trials [64]. While the bulk of research involves adult cancer cells or models, experiments employing bone/soft tissue sarcomas, brain and head and neck tumors, which could be diagnosed at any human age, could guide the corresponding research in pediatric malignancies, NPs-aided RT.

Photothermal therapy (PTT) supported by NPs represents a conventional method of applying heat and light to a nanodevice in order to promote the death of the cancer cells or the expression of specific antigens and markers [48]. AuNPs play a key role in photothermal therapy, due to their high light absorption and their ability to be tuned [15]. AuNPs have been used against gliomas in combination with PTT [15].

An interesting application was introduced by Cano-Mejia et al., oligodeoxynucleotide-coated Prussian blue nanoparticles (CpG-PBNPs) and PTT were combined with immunotherapy against neuroblastoma in mouse models [24]. Prussian blue is already an FDA-approved medication, including the pediatric population, against radioactive poisoning. The engineered CpG-PBNPs present photothermal properties, which were not affected by the CpG coating. The CpG works as an agonist for the toll-like receptor 9, increasing the CpG-PBNPs-based nanoimmunotherapy. The formulation was tested *in vitro* in neuroblastoma cell lines and *in vivo* in tumor-bearing mice. PTT was applied through a near-infrared laser. The goal was to achieve the contribution of the immune system to cell death, in order to both create immunological memory and eliminate distant cancer cells. The *in vivo* part of the experiment included 5 groups: vehicle, free CpG, CpG-PBNPs, PBNPs-PTT, CpG-PBNPs-PTT. The results demonstrated that the CpG-PBNPs-PTT had the best survival rates. The team also showed the generation of immunological memory by injecting the surviving mice with neuroblastoma cells and comparing their survival with a control group. The CpG-PBNP-PTT-treated mice had the best survival rates once again, indicating the presence of immunological memory.

Table 3 presents the discussed studies and their main results in pediatric cancer treatment supported by different types of NPs.

**Table 3:** Applications of NPs in pediatric cancer treatment

Type of NPs	Advantages	Type of therapy	Cancer type	Study results
AuNPs [51]	Targeted treatment Cross BBB Biocompatibility Low toxicity Easy functionalization Variety in sizes/shapes Stability High light absorption Ability to be tuned Photothermal properties	siRNA carrier	Ependymoma-MB	Silencing of the APE 1 gene results in lower expression of the repairing proteins. Combined with photon irradiation, it resulted in DNA damage and increased apoptosis of the cancer cells
LNPs [53,55]	Targeted treatment Biocompatibility Low toxicity Drug solubility Ideal nucleic acid vectors	siRNA carrier [53]	AML	Silencing of the oncogenic lncRNA LINC01257 results in a reduction in cancer cell proliferation and viability.
		Immunotherapy [55]	Glioma	Recession of the tumor growth. Regulation of the immunological axes and generation of immunological memory.
AFt Nanocage/ Liposome [57,59]	Targeted treatment Biocompatibility Low toxicity Drug solubility pH dependency Lipophilicity	Drug carrier	DIPG [57]	Sustained release and increased concentration of the drug inside the tumor in comparison with the free drug.
			Neuroblastoma [59]	Improved pharmacokinetics and increased accumulation inside the tumor, with a better anti-tumoral activity compared to the free drugs.
Micelle [58]	Targeted treatment Biocompatibility Low toxicity Amphophilic self-assemble		MB	Improved solubility and pharmacokinetics of the drug leading to a greater inhibition the tumor growth in comparison with the free drug.
CNDs [60]	Biocompatibility Low toxicity Water solubility High photoluminescence Adjustable structure		GBM	BBB crossing-cancer tissue targeting, limited effect on normal tissue (in contrast to the free drug)-cancer cells apoptosis
Squalenic acid [62]	Biocompatibility Ability to self-assemble		Osteosarcoma	Improved pharmacokinetics. Decreased cell proliferation compared to the free drugs.
PBNPs [24]	Biocompatibility Low toxicity Photothermal properties	PTT	Neuroblastoma	Increased survival in comparison with the control groups. Generation of immunological memory.
Polymeric NPs [54]	Potential biocompatibility Rapid degradation Easy functionalization Good immunogenic profile Ability to encapsulate DNA	DNA carrier	Teratoid/rhabdoid tumors	Activation of the ganciclovir prodrug, which induced cell killing. Increased survival of the treated population compared to the untreated.
Polymersome [56]	pH dependency	Immunotherapy	Neuroblastoma	Activation of the STING pathway, leading to immunological response and cell killing.

Note: BBB, Blood-Brain Barrier; siRNA, Small Interference Ribonucleic Acid; MB, Medulloblastoma; DNA, Deoxyribonucleic Acid; AML, Acute Myeloid Leukemia; lncRNA, long non-coding Ribonucleic Acid; DIPG, Diffuse Intrinsic Pontine Glioma; GBM, Glioblastoma; PTT, Photothermal Therapy; STING, Stimulator of the Interferon Genes.

#### 2.3.5 Clinical Trials

To the best of our knowledge, since 2019, 5 trials regarding pediatric cancer treatment assisted by NPs have been either completed or are recruiting. Investigating refractory solid tumors, sirolimus NPs combined with temozolomide/irinotecan (NCT02975882) [68] and vincristine combined with PEGylated liposomal doxorubicin (NCT04213612) [69] were studied to determine maximum tolerated dose (MTD), dose-limiting toxicities, and recommended phase 2 dose. In a similar direction, Mendez-Gomez et al. utilized RNA-LNP vaccines against newly diagnosed pediatric high-grade gliomas (NCT04573140) [55]. This phase 1 clinical trial aimed to identify the MTD. In the field of hematology, long-acting liposomal cytarabine combined with rituximab was investigated against mature B-cell Lymphoma (NCT01859819) [70]. A water-soluble nanoparticle formulation of Panobinostat, named MTX 110, combined with Gd, is investigated as a treatment for children with newly diagnosed diffuse midline glioma in a Phase 1 clinical trial (NCT04264143), which is currently being conducted [71].

Although the FDA has published its guidelines regarding NPs approval since 2005, the progress has been relatively slow, due to a variety of challenges [72]. The scalability and reproducibility are often difficult, while the final products require special conditions to ensure their stability, which increases the cost. Moreover, the characterization requires specific equipment, further raising the cost [72]. NPs, due to their small size and unique properties, like pH sensitivity, lipophilicity, or hydrophilicity, etc., have special pharmacokinetics and pharmacodynamics that have to be investigated thoroughly, especially for children. Pediatric patients may exhibit different reactions to NPs applications, while long-term accumulation of NPs in critical organs (liver, spleen, etc.) could result in further adverse effects. Up to date, Vyxeos is an anticancer nanomedicine, which has been approved for the pediatric population [12]. Vyxeos is a combination of two cytotoxic drugs, Daunorubicin and Cytarabine, encapsulated in an LNP [12]. The implementation of Vyxeos has shown significant clinical benefit in pediatric AML patients [12].

## 3 Discussion

NPs have emerged as a ray of hope for a lot of specialties, including pediatrics. Their applications vary greatly as they can improve and evolve a wide range of therapeutic and diagnostic procedures. NPs, due to their small size, can bypass the BBB and facilitate their accumulation inside the brain [73]. Owing to their easy functionalization and surface modification, they could overcome biological barriers that undermine the effectiveness of conventional cancer therapies and selectively target the tumor cells [73].

In the field of cancer diagnosis, biosensors have gained a lot of confidence over the last years as they speed up the diagnostic procedure, supporting fast screening and timely cancer detection [22,38,74]. Their sensitivity has improved to a great level since the integration of NPs.

Diagnostic imaging in pediatrics faces two great problems: the significant effect of radiation on the developing organism and the side effects of the contrast agents that are currently utilized. Since the imaging techniques require the cooperation and immobilization of the patients for a long period of time, children tend to receive anesthesia to undergo those tests. The longer half-time of ferumoxytol supports its use as a contrast agent, while the feed and sleep technique could be applied [6,7]. This novel contrast agent is associated with fewer side effects and provides better imaging results compared to the Gd-based one. Undoubtedly, both pediatricians and radiologists have to be trained properly to be able to incorporate it into the clinical routine.

NPs possess unique features that could be exploited in the field of pediatric cancer treatment with the intention of accomplishing a double goal: optimizing treatment schemes and reducing treatment-related side effects. NPs could serve as drug carriers; the targeted and modified drug release could provide increased intratumoral concentration, ensure that the medicine would act in a specific environment, and minimize its side effects [57,59,60]. Gene therapies could also utilize NPs as vehicles for the nucleic acids to establish efficient delivery in cancer cells [51,53]. Moreover, RT aided by metal and metal oxide NPs as radiosensitizers, could provide local dose enhancement in the irradiated target regions, increased cancer cells apoptosis and tumor growth inhibition without further increase of radiation-induced damages to healthy tissues and organs [65–67].

For the purpose of successfully widening the therapeutic ratio in pediatric cancer through the application of NPs, thorough research is needed. The differences between human and animal models dictate systematic analysis to determine the exact mechanisms of NPs’ biodistribution, clearance, and pharmacokinetics in children prior to clinical translation. The optimum administration methods in pediatric patients and NPs physicochemical characteristics should be investigated in depth, since they may affect NPs stability, their interaction with biomolecules, their biodistribution route, and the induced short-and long-term toxicity.

Nevertheless, the research faces some distinct obstacles. NPs complex manufacturing process and the high cost might restrict pre-clinical research. To overcome this, *in silico* approaches and Monte Carlo calculations could be initially used to offer a valuable insight into NPs-aided cancer therapy and guide the experimental translation *in vitro* and *in vivo*. However, the lack of pediatric models for the *in vivo* testing of the products raises further challenges [31]. The small number of cancer patients is an important limitation regarding the development of pediatric cancer models. Deep learning, though, could offer an alternative to strengthen the inputs of computational studies.

Additionally, NP based anticancer drugs already approved for adults cannot be translated in the pediatric population directly just by adjusting the dosage according to the child’s weight. Pharmacokinetics, pharmacodynamics, and immunological responses differ in children, while they also evolve and change during childhood. The side effects attributed to NPs usage could also vary and impact the developmental process of the pediatric patients. It is important to consider that NPs cross the BBB and could affect the brain, while long-term accumulation of NPs in tissues may also cause organ damage over time. Thus, the safety protocols have to be evaluated separately and thoroughly. Another important aspect regarding children is NPs’ administration. The majority of NPs are administered IV and since younger children do not tolerate injections, the implementation of such approach would be challenging. Finally, to the best of the authors’ knowledge, the interaction between NPs and RT in the pediatric population has not been investigated yet.

Nanomedicine is a relatively new domain, especially for pediatrics. The skepticism around it may hinder the clinical stages of the trials. This hesitation, which involves not only the parents but also the clinicians, could be suppressed through comprehensive efficiency and biosafety assessment studies combined with cost-effectiveness and regulatory analysis exclusively dedicated to the field of pediatric cancer care.

## 4 Conclusion

NPs open new horizons in cancer diagnosis and therapy in pediatric patients. NPs’ use in biosensor design could strengthen sensitivity, specificity, and allow for early cancer detection. Besides, NPs’ ability to target aggressive tumors and transfer successfully the contrast agents and chemotherapeutic drugs or genes, could offer the potential to improve both imaging accuracy and treatment efficacy. Moreover, high-Z NPs could serve as radiosensitizers to optimize the RT the patients receive, in terms of enhancing tumor control probability without further radiation-related side effects. Thus, the integration of NPs in pediatric oncology may improve both the survival rates, while enhancing the quality of life for the young patients. However, it should be noted that further systematic, consistent, interdisciplinary research is needed to ensure NPs’ efficiency and long-term safety in order to incorporate them into the therapeutic protocols.

## Data Availability

Not applicable.

## Abbreviations

^18^F-FDG: ^18^F-fluorodeoxyglucose
AFt: Apoferritin
AML: Acute Myeloid Leukemia
ARG: L-arginine
AuNPs: Gold Nanoparticles
BBB: Blood-Brain Barrier
Bcl: B-cell lymphoma
CED: Conventional Enhanced Delivery
CNDs: Carbon Nitrate dots
CNS: Central Nervous System
CSCs: Cancer Stem Cells
DHEAS: Dehydroepiandrosterone Sulfate
DIPG: Diffuse Intrinsic Pontine Glioma
EPR: Enhanced Permeability and Retention
EVs: Extracellular Vehicles
FDA: Food and Drug Administration
FNH: Focal Nodular Hyperplasia
FeO: Iron Oxide
FNH: Focal nodular hyperplasia
GBM: Glioblastoma
Gd: Gadolinium
GM: Gemcitabine
HfO: Hafnium Oxide
HIV: Human Immunodeficiency Virus
HSVtK: Herpes Simplex Virus Type I Thymidine Kinase
IV: Intravenous
IONPs: Iron Oxide Nanoparticles
lncRNA: long non-coding RNA
LN: Lymph Node
LNP: Lipid Nanoparticle
mTOR: Mammalian Target Of Rapamycin
MTD: Maximum Tolerated Dose
MB: Medulloblastoma
miRNA: Micro RNA
MRI: Magnetic Resonance Imaging
NPs: Nanoparticles
PBAEs: Poly(beta-amino ester)s
PBNPs: Prussian Blue NPs
PET: Positron Emission Tomography
PTPmu: Protein Tyrosine Phosphatase mu
PTT: Photothermal Therapy
PZQ: Praziquantel
RES: Reticuloendothelial System
RT: Radiotherapy
siRNA: Small interference RNA
SQ-GM: Squalenoyl-gemcitabine
SPARC: Secreted Protein Acidic-Cysteine
STING: Stimulator of the Interferon Genes
T2WI: T2-Weighted Images
TMZ: Temozolomide
Tf: Transferrin protein
VOCs: Volatile Organic Compounds

## References

[1] Siegel RL, Kratzer TB, Giaquinto AN, Sung H, Jemal A. Cancer statistics, 2025. CA Cancer J Clin. 2025;75(1):10–45; 39817679 10.3322/caac.21871 PMC11745215

[2] Steliarova-Foucher E, Colombet M, Ries LAG, Moreno F, Dolya A, Bray F, et al. International incidence of childhood cancer, 2001–10: a population-based registry study. Lancet Oncol. 2017;18(6):719–31; 28410997 10.1016/S1470-2045(17)30186-9 PMC5461370

[3] Lam CG, Howard SC, Bouffet E, Pritchard-Jones K. Science and health for all children with cancer. Science. 2019;363(6432):1182–6. doi:10.1126/science.aaw4892; 30872518

[4] Ferlay J, Colombet M, Soerjomataram I, Parkin DM, Piñeros M, Znaor A, et al. Cancer statistics for the year 2020: an overview. Int J Cancer. 2021;149(4):778–89. doi:10.1002/ijc.33588; 33818764

[5] Mullen CJR, Barr RD, Franco EL. Timeliness of diagnosis and treatment: the challenge of childhood cancers. Br J Cancer. 2021;125(12):1612–20. doi:10.1038/s41416-021-01533-4; 34471259 PMC8651632

[6] Adams LC, Jayapal P, Ramasamy SK, Morakote W, Yeom K, Baratto L, et al. Ferumoxytol-enhanced MRI in children and young adults: state of the art. Am J Roentgenol. 2023;220(4):590–603. doi:10.2214/ajr.22.28453; 36197052 PMC10038879

[7] Lai LM, Cheng JY, Alley MT, Zhang T, Lustig M, Vasanawala SS. Feasibility of ferumoxytol-enhanced neonatal and young infant cardiac MRI without general anesthesia. J Magn Reson Imaging. 2017;45(5):1407–18. doi:10.1002/jmri.25482; 27678106 PMC5373922

[8] Kanda T, Oba H, Toyoda K, Kitajima K, Furui S. Brain gadolinium deposition after administration of gadolinium-based contrast agents. Jpn J Radiol. 2016;34(1):3–9. doi:10.1007/s11604-015-0503-5; 26608061

[9] Siedek F, Muehe AM, Theruvath AJ, Avedian R, Pribnow A, Spunt SL, et al. Comparison of ferumoxytol-and gadolinium chelate-enhanced MRI for assessment of sarcomas in children and adolescents. Eur Radiol. 2020;30(3):1790–803. doi:10.1007/s00330-019-06569-y; 31844962 PMC7035151

[10] Yang S, Wallach M, Krishna A, Kurmasheva R, Sridhar S. Recent developments in nanomedicine for pediatric cancer. J Clin Med. 2021;10(7):1437. doi:10.3390/jcm10071437; 33916177 PMC8036287

[11] Gavas S, Quazi S, Karpiński TM. Nanoparticles for cancer therapy: current progress and challenges. Nanoscale Res Lett. 2021;16(1):173. doi:10.1186/s11671-021-03628-6; 34866166 PMC8645667

[12] Yang S, Aggarwal K, Jurczyszak J, Brown N, Sridhar S. Nanomedicine therapies for pediatric diseases. Wiley Interdiscip Rev. 2024;16(5):e1996. doi:10.1002/wnan.1996; 39420230 PMC11493394

[13] Daneman R, Prat A. The blood–brain barrier. Cold Spring Harb Perspect Biol. 2015;7(1):a020412; 25561720 10.1101/cshperspect.a020412 PMC4292164

[14] Dracham CB, Shankar A, Madan R. Radiation induced secondary malignancies: a review article. Radiat Oncol J. 2018;36(2):85–94. doi:10.3857/roj.2018.00290; 29983028 PMC6074073

[15] Guido C, Baldari C, Maiorano G, Mastronuzzi A, Carai A, Quintarelli C, et al. Nanoparticles for diagnosis and target therapy in pediatric brain cancers. Diagnostics. 2022;12(1):173. doi:10.3390/diagnostics12010173; 35054340 PMC8774904

[16] Abballe L, Spinello Z, Antonacci C, Coppola L, Miele E, Catanzaro G, et al. Nanoparticles for drug and gene delivery in pediatric brain tumors’ cancer stem cells: current knowledge and future perspectives. Pharmaceutics. 2023;15(2):505. doi:10.3390/pharmaceutics15020505; 36839827 PMC9962005

[17] Pozzi M, Jonak Dutta S, Kuntze M, Bading J, Rüßbült JS, Fabig C, et al. Visualization of the high surface-to-volume ratio of nanomaterials and its consequences. J Chem Educ. 2024;101(8):3146–55. doi:10.1021/acs.jchemed.4c00089; 39157433 PMC11328123

[18] Augustine R, Hasan A, Primavera R, Wilson RJ, Thakor AS, Kevadiya BD. Cellular uptake and retention of nanoparticles: insights on particle properties and interaction with cellular components. Mater Today Commun. 2020;25:101692. doi:10.1016/j.mtcomm.2020.101692.

[19] Mitchell MJ, Billingsley MM, Haley RM, Wechsler ME, Peppas NA, Langer R. Engineering precision nanoparticles for drug delivery. Nat Rev Drug Discov. 2021;20(2):101–24. doi:10.1038/s41573-020-0090-8; 33277608 PMC7717100

[20] Sell M, Lopes AR, Escudeiro M, Esteves B, Monteiro AR, Trindade T, et al. Application of nanoparticles in cancer treatment: a concise review. Nanomaterials. 2023;13(21):2887. doi:10.3390/nano13212887; 37947732 PMC10650201

[21] Mundekkad D, Cho WC. Nanoparticles in clinical translation for cancer therapy. Int J Mol Sci. 2022;23(3):1685; 35163607 10.3390/ijms23031685 PMC8835852

[22] Sun C, Xiao F, Fu J, Huang X, Jia N, Xu Z, et al. Loop-mediated isothermal amplification coupled with nanoparticle-based lateral biosensor for rapid sensitive and specific detection of *Bordetella pertussis*. Front Bioeng Biotechnol. 2022;9:797957. doi:10.3389/fbioe.2021.797957; 35211469 PMC8861531

[23] Joudeh N, Linke D. Nanoparticle classification, physicochemical properties, characterization, and applications: a comprehensive review for biologists. J Nanobiotechnol. 2022;20(1):262. doi:10.1186/s12951-022-01477-8; 35672712 PMC9171489

[24] Cano-Mejia J, Bookstaver ML, Sweeney EE, Jewell CM, Fernandes R. Prussian blue nanoparticle-based antigenicity and adjuvanticity trigger robust antitumor immune responses against neuroblastoma. Biomater Sci. 2019;7(5):1875–87. doi:10.1039/c8bm01553h; 30789175 PMC6491208

[25] Kievit FM, Stephen ZR, Wang K, Dayringer CJ, Sham JG, Ellenbogen RG, et al. Nanoparticle mediated silencing of DNA repair sensitizes pediatric brain tumor cells to γ-irradiation. Mol Oncol. 2015;9(6):1071–80. doi:10.1016/j.molonc.2015.01.006; 25681012 PMC4439369

[26] Arif M, Nawaz AF, Ullah khan S, Mueen H, Rashid F, Hemeg HA, et al. Nanotechnology-based radiation therapy to cure cancer and the challenges in its clinical applications. Heliyon. 2023;9(6):e17252. doi:10.1016/j.heliyon.2023.e17252; 37389057 PMC10300336

[27] Rashidi A, Baratto L, Theruvath AJ, Greene EB, Jayapal P, Hawk KE, et al. Improved detection of bone metastases in children and young adults with ferumoxytol-enhanced MRI. Radiology. 2023;5(2):e220080. doi:10.1148/rycan.220080; 36999999 PMC10077085

[28] Muehe AM, Siedek F, Theruvath AJ, Seekins J, Spunt SL, Pribnow A, et al. Differentiation of benign malignant lymph nodes in pediatric patients on ferumoxytol-enhanced PET/MRI. Theranostics. 2020;10(8):3612–21. doi:10.7150/thno.40606; 32206111 PMC7069081

[29] Khurana A, Nejadnik H, Gawande R, Guiting Lin M, Lee S, Solomon Messing M, et al. Intravenous ferumoxytol allows noninvasive MR imaging monitoring of macrophage migration into stem cell transplants. Radiology. 2012;264(3):803–11. doi:10.1148/radiol.12112393; 22820731 PMC3426856

[30] Huang Y, Singer TG, Iv M, Lanzman B, Nair S, Stadler JA, et al. Ferumoxytol-enhanced MRI for surveillance of pediatric cerebral arteriovenous malformations. J Neurosurg Pediatr. 2019;24(4):407–14. doi:10.3171/2019.5.peds1957; 31323627

[31] Covarrubias G, Johansen ML, Vincent J, Erokwu BO, Craig SEL, Rahmy A, et al. TPmu-targeted nanoparticles label invasive pediatric and adult glioblastoma. Nanomedicine. 2020;28:102216; 32413511 10.1016/j.nano.2020.102216 PMC7573884

[32] Botti G, Dalpiaz A, Pavan B. Targeting systems to the brain obtained by merging prodrugs, nanoparticles, and nasal administration. Pharmaceutics. 2021;13(8):1144. doi:10.3390/pharmaceutics13081144; 34452105 PMC8399330

[33] Lenzen A, Garcia Sosa RM, Habiby R, DiPatri AJ Jr, Pillay Smiley N. Pediatric central nervous system tumor diagnosis, complications, and emergencies. Clin Pediatr Emerg Med. 2018;19(2):153–61. doi:10.1016/j.cpem.2018.06.003.

[34] Dorfman RE, Alpern MB, Gross BH, Sandier MA. Upper abdominal lymph nodes: criteria for normal size determined with CT. Radiology. 1991;180(2):319–22. doi:10.1148/radiology.180.2.2068292; 2068292

[35] Theruvath AJ, Rashidi A, Nyalakonda RR, Avedian RS, Steffner RJ, Spunt SL, et al. Ferumoxytol magnetic resonance imaging detects joint and pleural infiltration of bone sarcomas in pediatric and young adult patients. Pediatr Radiol. 2021;51(13):2521–9. doi:10.1007/s00247-021-05156-y; 34410452 PMC8602726

[36] Toth GB, Varallyay CG, Horvath A, Bashir MR, Choyke PL, Daldrup-Link HE, et al. Current and potential imaging applications of ferumoxytol for magnetic resonance imaging. Kidney Int. 2017;92(1):47–66. doi:10.1016/j.kint.2016.12.037; 28434822 PMC5505659

[37] Shahid M, Albergo N, Purvis T, Heron K, Gaston L, Carter S, et al. Management of sarcomas possibly involving the knee joint when to perform extra-articular resection of the knee joint and is it safe? Eur J Surg Oncol. 2017;43(1):175–80. doi:10.1016/j.ejso.2016.05.018; 27266818

[38] Mohajeri S, Moayedi S, Azimi L, Akrami M, Rad-Malekshahi M, Fazeli MR, et al. Nanobiosensor based on sugar code-aunps aggregation: a key to opening new gates in rapid diagnosis of streptococcal pharyngitis. Front Bioeng Biotechnol. 2022;10:957271. doi:10.3389/fbioe.2022.957271; 35935503 PMC9354983

[39] Lima D, Inaba J, Clarindo Lopes L, Calaça GN, Los Weinert P, Lenzi Fogaça R, et al. Label-free impedimetric immunosensor based on arginine-functionalized gold nanoparticles for detection of DHEAS, a biomarker of pediatric adrenocortical carcinoma. Biosens Bioelectron. 2019;133:86–93. doi:10.1016/j.bios.2019.02.063; 30909017

[40] Fay AP, Elfiky A, Teló GH, McKay RR, Kaymakcalan M, Nguyen PL, et al. Adrenocortical carcinoma: the management of metastatic disease. Crit Rev Oncol Hematol. 2014;92(2):123–32. doi:10.1016/j.critrevonc.2014.05.009; 24958272 PMC4578298

[41] Bordbar MM, Barzegar H, Tashkhourian J, Bordbar M, Hemmateenejad B. A non-invasive tool for early detection of acute leukemia in children using a paper-based optoelectronic nose based on an array of metallic nanoparticles. Anal Chim Acta. 2021;1141:28–35. doi:10.1016/j.aca.2020.10.029; 33248659

[42] Tang H, Lu Y, Zhang L, Wu Z, Hou X, Xia H. Determination of volatile organic compounds exhaled by cell lines derived from hematological malignancies. Biosci Rep. 2017;37(3):BSR20170106. doi:10.1042/bsr20170106; 28507202 PMC5479021

[43] Wu M, Neilson A, Swift AL, Moran R, Tamagnine J, Parslow D, et al. Multiparameter metabolic analysis reveals a close link between attenuated mitochondrial bioenergetic function and enhanced glycolysis dependency in human tumor cells. Am J Physiol Cell Physiol. 2007;292:125–36. doi:10.1152/ajpcell.00247.2006; 16971499

[44] Rashidi N, Davidson M, Apostolopoulos V, Nurgali K. Nanoparticles in cancer diagnosis and treatment: progress challenges, and opportunities. J Drug Deliv Sci Technol. 2024;95:105599. doi:10.1016/j.jddst.2024.105599.

[45] Claude F, Ubertini G, Szinnai G. Endocrine disorders in children with brain tumors: at diagnosis after surgery radiotherapy and chemotherapy. Children. 2022;9(11):1617. doi:10.3390/children9111617; 36360345 PMC9688119

[46] Otth M, Wyss J, Scheinemann K. Long-term follow-up of pediatric CNS tumor survivors—a selection of relevant long-term issues. Children. 2022;9(4):447. doi:10.3390/children9040447; 35455491 PMC9029633

[47] Löscher W, Potschka H. Drug resistance in brain diseases and the role of drug efflux transporters. Nat Rev Neurosci. 2005;6(8):591–602. doi:10.1038/nrn1728; 16025095

[48] El Moukhtari SH, Garbayo E, Fernández-Teijeiro A, Rodríguez-Nogales C, Couvreur P, Blanco-Prieto MJ. Nanomedicines and cell-based therapies for embryonal tumors of the nervous system. J Control Release. 2022;348:553–71. doi:10.1016/j.jconrel.2022.06.010; 35705114

[49] Zhang P, Darmon A, Marill J, Anesary NM, Paris S. Radiotherapy-activated hafnium oxide nanoparticles produce abscopal effect in a mouse colorectal cancer model. Int J Nanomed. 2020;15:3843–50. 10.2147/IJN.S250490 PMC7280060 32581534

[50] Chen Y, Yang J, Fu S, Wu J. Gold nanoparticles as radiosensitizers in cancer radiotherapy. Int J Nanomed. 2020;15:9407–30. 10.2147/IJN.S272902 PMC7699443 33262595

[51] Liu Z, Yan H, Li H. Silencing of DNA repair sensitizes pediatric brain tumor cells to γ-irradiation using gold nanoparticles. Environ Toxicol Pharmacol. 2017;53:40–5. doi:10.1016/j.etap.2017.04.017; 28501783

[52] Arun G, Diermeier SD, Spector DL. Therapeutic targeting of long non-coding RNAs in cancer. Trends Mol Med. 2018;24:257–77. doi:10.1016/j.molmed.2018.01.001; 29449148 PMC5840027

[53] Connerty P, Moles E, de Bock CE, Jayatilleke N, Smith JL, Meshinchi S, et al. Development of siRNA-loaded lipid nanoparticles targeting long non-coding RNA LINC01257 as a novel and safe therapeutic approach for t(8;21) pediatric acute myeloid leukemia. Pharmaceutics. 2021;13(10):1681. doi:10.3390/pharmaceutics13101681; 34683974 PMC8539450

[54] Choi J, Rui Y, Kim J, Gorelick N, Wilson DR, Kozielski K, et al. Nonviral polymeric nanoparticles for gene therapy in pediatric CNS malignancies. Nanomedicine. 2020;23:102115. doi:10.1016/j.nano.2019.102115; 31655205 PMC7027378

[55] Mendez-Gomez H, McGuiness J, Grippin A, Weidert F, Carrera-Justiz S, Mitchell D, et al. Customizable multi-lamellar RNA-nanoparticles for pediatric glioma. Neuro-Oncol. 2021;23(1):i29. doi:10.1093/neuonc/noab090.120.

[56] Wang-Bishop L, Wehbe M, Shae D, James J, Hacker BC, Garland K, et al. Potent STING activation stimulates immunogenic cell death to enhance antitumor immunity in neuroblastoma. J Immunother Cancer. 2020;8(1):e000282. doi:10.1136/jitc-2019-000282; 32169869 PMC7069313

[57] Heravi Shargh V, Luckett J, Bouzinab K, Paisey S, Turyanska L, Singleton WGB, et al. Chemosensitization of temozolomide-resistant pediatric diffuse midline glioma using potent nanoencapsulated forms of a N(3)-propargyl analogue. ACS Appl Mater Interfaces. 2021;13(30):35266–80. doi:10.1021/acsami.1c04164; 34310112

[58] Infante P, Malfanti A, Quaglio D, Balducci S, De Martin S, Bufalieri F, et al. Glabrescione B delivery by self-assembling micelles efficiently inhibits tumor growth in preclinical models of Hedgehog-dependent medulloblastoma. Cancer Lett. 2021;499:220–31. doi:10.1016/j.canlet.2020.11.028; 33249196

[59] Yang X, Tong J, Guo L, Qian Z, Chen Q, Qi R, et al. Bundling potent natural toxin cantharidin within platinum (IV) prodrugs for liposome drug delivery and effective malignant neuroblastoma treatment. Nanomedicine. 2017;13(1):287–96. doi:10.1016/j.nano.2016.08.024; 27591962

[60] Liyanage PY, Zhou Y, Al-Youbi AO, Bashammakh AS, El-Shahawi MS, Vanni S, et al. Pediatric glioblastoma target-specific efficient delivery of gemcitabine across the blood-brain barrier: via carbon nitride dots. Nanoscale. 2020;12(14):7927–38. doi:10.1039/d0nr01647k; 32232249

[61] Rodríguez-Nogales C, Sebastián V, Irusta S, Desmaële D, Couvreur P, Blanco-Prieto MJ. A unique multidrug nanomedicine made of squalenoyl-gemcitabine and alkyl-lysophospholipid edelfosine. Eur J Pharm Biopharm. 2019;144:165–73. doi:10.1016/j.ejpb.2019.09.017; 31546021

[62] Rodríguez-Nogales C, Mura S, Couvreur P, Blanco-Prieto MJ. Squalenoyl-gemcitabine/edelfosine nanoassemblies: anticancer activity in pediatric cancer cells and pharmacokinetic profile in mice. Int J Pharm. 2020;582:119345. doi:10.1016/j.ijpharm.2020.119345; 32311470

[63] Carvalho AM, Greene MK, Smyth P, Mutch A, McLaughlin KM, Cairns LV, et al. Development of CD33-targeted dual drug-loaded nanoparticles for the treatment of pediatric acute myeloid leukemia. Biomacromolecules. 2024;25(10):6503–14. doi:10.1021/acs.biomac.4c00672; 39235263 PMC11480974

[64] Xiao S, Wang X, Chen B, Mu M, Han B, Chen N, et al. Enhancing tumor radiotherapy sensitivity through metal nanomaterials: a comprehensive review. Malig Spectr. 2024;1(4):243–62. doi:10.1002/msp2.52.

[65] Zhang X, Wang H, Coulter JA, Yang R. Octaarginine-modified gold nanoparticles enhance the radiosensitivity of human colorectal cancer cell line LS180 to megavoltage radiation. Int J Nanomed. 2018;13:3541–52. doi:10.2147/ijn.s161157; 29950834 PMC6016276

[66] Jia TT, Yang G, Mo SJ, Wang ZY, Li BJ, Ma W, et al. Atomically precise gold-levonorgestrel nanocluster as a radiosensitizer for enhanced cancer therapy. ACS Nano. 2019;13(7):8320–8. doi:10.1021/acsnano.9b03767; 31241895

[67] Vlastou E, Pantelis E, Efstathopoulos EP, Karaiskos P, Kouloulias V, Platoni K. Quantification of nanoscale dose enhancement in gold nanoparticle-aided external photon beam radiotherapy. Cancers. 2022;14(9):2167. doi:10.3390/cancers14092167; 35565296 PMC9102439

[68] Cramer SL, Reddy AT, Minard CG, Voss S, Fox E, Liu X, et al. A phase 1 study of ABI-009 (nab-sirolimus) in combination with temozolomide and irinotecan in pediatric patients with recurrent or refractory solid tumors, including CNS tumors—a children’s oncology group pediatric early phase clinical trial network study ADVL1514. Cancer Med. 2024;13(21):e70376. doi:10.1200/jco.2022.40.16_suppl.10022. 39487711 PMC11533328

[69] Lu S, Wang J, Huang J, Sun F, Zhu J, Que Y, et al. Pegylated liposomal doxorubicin combined with cyclophosphamide and vincristine in pediatric patients with relapsed/refractory solid tumor: a single-arm, open-label, phase I study. eClinicalMedicine. 2024;73:102701. doi:10.1016/j.eclinm.2024.102701; 39007065 PMC11246015

[70] Goldman S, Barth M, Shiramizu B, Shi Q, Hochberg J, Klejmont L, et al. A dose substitution of anthracycline intensity with dose-dense rituximab in children and adolescents with good-risk mature B-cell lymphoma. Leukemia. 2021;35(10):2994–7. doi:10.1038/s41375-021-01256-8; 33941850 PMC12582166

[71] Szalontay L, CreveCoeur T, Neira J, Englander Z, Spinazzi E, Canoll P, et al. A phase I study examining the feasibility of intermittent convection-enhanced delivery (CED) of MTX110 for the treatment of children with newly diagnosed diffuse midline gliomas. Neuro-Oncol. 2024;26(Supplement_4). doi:10.1093/neuonc/noae064.648.

[72] Omidian H, Mfoafo K. Exploring the potential of nanotechnology in pediatric healthcare: advances, challenges, and future directions. Pharmaceutics. 2023;15(6):1583. doi:10.3390/pharmaceutics15061583; 37376032 PMC10303369

[73] Lu Q, Yu H, Zhao T, Zhu G, Li X. Nanoparticles with transformable physicochemical properties for overcoming biological barriers. Nanoscale. 2023;15:13202–23; 37526946 10.1039/d3nr01332d

[74] Wu X, Wang Y, Yin Q, Jiao W, Sun L, Qi H, et al. A diagnostic test that uses isothermal amplification and lateral flow detection sdaA can detect tuberculosis in 60 min. J Appl Microbiol. 2021;130(6):2102–10. doi:10.1111/jam.14902; 33070404

